# Chinese herbal medicine and active ingredients for diabetic cardiomyopathy: molecular mechanisms regulating endoplasmic reticulum stress

**DOI:** 10.3389/fphar.2023.1290023

**Published:** 2023-11-07

**Authors:** Lianjun Ao, Zhengtao Chen, Jiacheng Yin, Yulin Leng, Yue Luo, Xiaoxu Fu, Hanyu Liu, Xiaoke Liu, Hong Gao, Chunguang Xie

**Affiliations:** ^1^ Hospital of Chengdu University of Traditional Chinese Medicine, Chengdu, Sichuan, China; ^2^ Affiliated Hospital of Jiangxi University of Chinese Medicine, Nanchang, Jiangxi, China; ^3^ TCM Regulating Metabolic Diseases Key Laboratory of Sichuan Province, Chengdu, Sichuan, China; ^4^ Department of Endocrinology, Hospital of Chengdu University of Traditional Chinese Medicine, Chengdu, China

**Keywords:** diabetic cardiomyopathy, endoplasmic reticulum stress, Chinese herbal medicine, active ingredients, mechanism of action

## Abstract

**Abstract Background:** Diabetic cardiomyopathy (DCM) is one of the serious microvascular complications of diabetes mellitus. It is often associated with clinical manifestations such as arrhythmias and heart failure, and significantly reduces the quality of life and years of survival of patients. Endoplasmic reticulum stress (ERS) is the removal of unfolded and misfolded proteins and is an important mechanism for the maintenance of cellular homeostasis. ERS plays an important role in the pathogenesis of DCM by causing cardiomyocyte apoptosis, insulin resistance, calcium imbalance, myocardial hypertrophy and fibrosis. Targeting ERS is a new direction in the treatment of DCM. A large number of studies have shown that Chinese herbal medicine and active ingredients can significantly improve the clinical outcome of DCM patients through intervention in ERS and effects on myocardial structure and function, which has become one of the hot research directions.

**Purpose:** The aim of this review is to elucidate and summarize the roles and mechanisms of Chinese herbal medicine and active ingredients that have the potential to modulate endoplasmic reticulum stress, thereby contributing to better management of DCM.

**Methods:** Databases such as PubMed, Web of Science, China National Knowledge Internet, and Wanfang Data Knowledge Service Platform were used to search, analyze, and collect literature, in order to review the mechanisms by which phytochemicals inhibit the progression of DCM by targeting the ERS and its key signaling pathways. Keywords used included “diabetic cardiomyopathy” and “endoplasmic reticulum stress.”

**Results:** This review found that Chinese herbs and their active ingredients can regulate ERS through IRE1, ATF6, and PERK pathways to reduce cardiomyocyte apoptosis, ameliorate myocardial fibrosis, and attenuate myocardial hypertrophy for the treatment of DCM.

**Conclusion:** A comprehensive source of information on potential ERS inhibitors is provided in this review. The analysis of the literature suggests that Chinese herbal medicine and its active ingredients can be used as potential drug candidates for the treatment of DCM. In short, we cannot ignore the role of traditional Chinese medicine in regulating ERS and treating DCM, and look forward to more research and new drugs to come.

## 1 Introduction

Diabetic cardiomyopathy (DCM) is a serious diabetic microvascular complication in the absence of other cardiac risk factors (e.g., coronary artery disease, hypertension, significant valvular disease, etc.) in which there are structural and functional abnormalities of the heart muscle (Jia et al., 2018). The name of the disease was first proposed by S Rubler in 1972 (Rub et al., 1972). There are 2 phenotypes of DCM, restrictive and dilated, both of which are manifestations of heart failure, with the difference being whether the ejection fraction is reduced or not (Macvanin et al., 2023). The European Society of Cardiology (Seferovic et al., 2018) and the American Heart Association (Dunlay et al., 2019) state that type 2 diabetes mellitus (T2DM) often coincides with heart failure, and that the prevalence of heart failure is higher in patients with type 2 diabetes mellitus compared to the general population, at approximately 10%–30%.

At present, Western medical treatment mainly includes lifestyle intervention, blood sugar control, lipid regulation, and anti-heart failure, but long-term drug treatment may lead to drug dependence, impaired liver and kidney function, and other adverse effects, and there are no specific drugs for DCM in Western medicine. And the risk of heart failure may be increased with traditional hypoglycemic drugs such as sulfonylureas and thiazolidinediones (Nakamura et al., 2022). Therefore, exploring traditional chinese medicine (TCM) treatment options for DCM is of positive significance. Several clinical studies have shown that TCM may be effective in the prevention and treatment of DCM in terms of clinical efficacy and symptom improvement (Lu and Zhou, 2010; Du et al., 2018; Shi et al., 2020; Zhang KW. et al., 2022; Niu et al., 2022; Tan et al., 2022). In a randomized controlled study of 82 patients with DCM, the experimental group was treated with Tongluo Nourishing Yin Formula + basic treatment, and the control group was treated with basic treatment. The results showed that the traditional Chinese medicine formula Tongluo Nourishing Yin Formula could improve clinical efficacy and left ventricular ejection fraction (LVEF), left ventricular end diastolic diameter (LVEDD), left ventricular end systolic diameter (LVESD), and left ventricular end systolic diameter (LVEDD) of the patients with DCM. Left ventricular ejection fraction (LVEF), left ventricular end diastolic diameter (LVEDD), left ventricular end systolic diameter (LVESD) cardiac function indexes in DCM patients (Zhang KW. et al., 2022). Benefiting qi-nourishing yin-activating blood circulation formula improved the left ventricular diastolic function, regulated blood glucose, improved the antioxidant capacity of the body, improved the clinical symptoms of the patients, and had a certain preventive effect on the cardiomyopathy caused by diabetes mellitus in the elderly (Lu and Zhou, 2010). A total of 33 RCT studies were included in a meta-analysis of the effects of anthocyanins on cardiometabolic health, which showed that the natural compound anthocyanins can lower blood glucose, blood lipids, and have cardiovascular protective effects (Yang et al., 2017). Many researchers have been involved in order to elucidate the mechanism of action of TCM in the treatment of DCM. It has been demonstrated that the active ingredients of Chinese medicines (Resveratrol (Fang et al., 2022), Daidzein (Laddha and Kulkarni, 2021), Salvianolic acid A (Gong et al., 2023), Rhynchophylline (Liu et al., 2022), Lycium barbarum polysaccharide (Liu et al., 2019), Salidroside (Li et al., 2021), Piperine (Wang et al., 2020), Quercetin (Jiang et al., 2022), Scutellarin (Xu et al., 2021), Luteolin (Li et al., 2019), etc.), herbal monomers (pomegranate peel (Abo-Saif et al., 2023), cinnamon (Kumar et al., 2022), galangin (Abukhalil et al., 2021), etc.), and herbal compound formulas (Fufang Xueshuantong (Peng et al., 2022), Fufang Zhenzhu Tiao Zhi formula (Yan et al., 2022; Yan et al., 2023), Si-Miao-Yong-An decoction (Li et al., 2020), Yunpi-Huoxue-Sanjie formula (Zhang et al., 2022), etc.) can treat DCM through anti-oxidative stress, anti-inflammation, anti-apoptosis, anti-fibrosis, regulation of autophagy, regulation of calcium homeostasis, regulation of cardiac lipotoxicity, and restoration of mitochondrial function.In further mechanistic studies, endoplasmic reticulum stress (ERS) was found to be a key target for treating DCM in TCM.

ERS is crucial in the development of DCM, inducing apoptosis, affecting myocardial autophagy and aggravating DCM, which is one of the important molecular mechanisms of DCM(1). Research has demonstrated that blocking the ERS pathway can decrease ERS, alleviate apoptosis, inflammation, and cardiac dysfunction in DCM myocytes (Nam et al., 2015), which is a potential target for the treatment of DCM. In recent years, the regulation of ERS has become a research hotspot for the prevention and treatment of DCM. A large number of studies have confirmed that Chinese medicine can prevent and treat DCM through the regulation of ERS, but there is still no review study in this direction. Therefore, this paper focuses on the regulation of ERS by TCM to improve DCM. It also provides an overview for further research, clinical application, and development of innovative TCM drugs for the prevention and treatment of DCM. In the study of ERS modulation by TCM, it was found that the active ingredients of TCM [Paeonol (Choy et al., 2016), Oridonin (Zhou et al., 2023), Lycopene (Sun et al., 2022), Salidroside (Wang et al., 2017), Coptisine (Zhou et al., 2021), Mangiferin (Li et al., 2020)] and TCM compound formulas [Compound Tongluo Decoction (Hui et al., 2022), Dahuang Danshen Decoction (Lian et al., 2021), Fuzheng Nizeng Decoction (Chu et al., 2022), Jiedutongluotiaogan formula (Luo et al., 2022), Qishen Granule (Zhang et al., 2020), Tong-Xie-Yao-Fang (Zhang et al., 2022)] can from different pathways regulation of ERS.

We searched Pubmed, Web of science and Chinese National Knowledge Infrastructure (CNKI) for domestic and foreign studies. The search key words are “Endoplasmic Reticulum Stresses”, “diabetic cardiomyopathy.”

## 2 Endoplasmic reticulum stress

**FIGURE 1:**
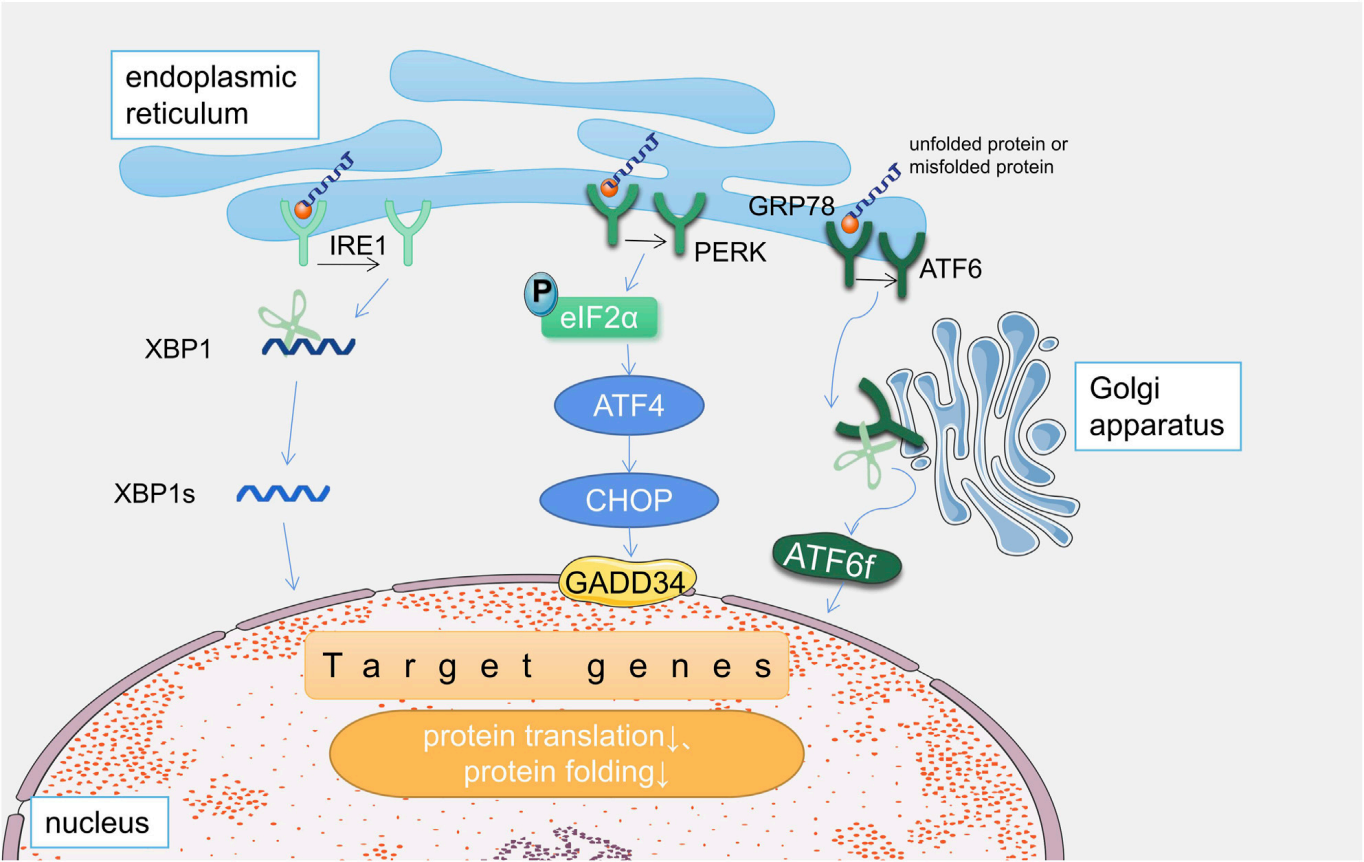
Three pathways of unfolded protein response in endoplasmic reticulum stress.

The endoplasmic reticulum (ER) is an organelle in the cytoplasm of eukaryotic cells, whose functions are mainly related to protein synthesis, including post-translational modification, folding, sorting (Sanvictores and Davis, 2022). Many factors can interfere with ER function, causing unfolded or misfolded proteins to accumulate in the ER, called endoplasmic reticulum stress. When unfolded or misfolded proteins accumulate excessively in the ER lumen, an ER-specific adaptive program--the unfolded protein response (UPR), is activated to increase the degradation of these misfolded proteins to maintain protein homeostasis (Guzel et al., 2017). There are three sensors that activate the UPR (as shown in Figure 1), including inositol-requiring factor 1 (IRE1), activated transcription factor 6 (ATF6), and protein kinase R-like endoplasmic reticulum kinase (PERK). Which are located on the endoplasmic reticulum membrane. Under basal conditions, the intraluminal structures of IRE1, ATF6 and PERK are inactive when bound to the ERS chaperone molecule glucose regulatory protein 78 (GRP78), also known as binding immunoglobulin (BIP).When the levels of unfolded or misfolded proteins are elevated, the three proteins mentioned above dissociate from GRP78, which then becomes activated and activates downstream signaling molecules (Frakes and Dillin, 2017).

In the IRE1 pathway, its heterodimer IRE1α cleavages X-box binding protein 1 (XBP1) mRNA to generate XBP1S, which degrades the misfolded protein; in addition, IRE1 activates the apoptosis pathway to promotes apoptosis (Yang et al., 2015). In the PERK pathway, activated PERK phosphorylates the eukaryotic translation initiation factor 2 alpha (eIF2α), which prevents the translation of most proteins and reduces the burden of protein folding on the ER (He et al., 2018); It also activates activating transcription factor 4 (ATF4), induces the production of C/EBP homologous protein/growth arrest and DNA damage (CHOP/GADD), and regulates protein translation (Lakshmanan et al., 2013; Younce et al., 2013; Yang et al., 2015). In addition, it can phosphorylate nuclear factor E2-related factor 2 (NRF2), which affects oxidative stress (Yang et al., 2015). In the ATF6 pathway, activation of ERS leads to translocation of ATF6 to the Golgi, cleavage, activation, and release as a 50 kDa amino-terminal cytoplasmic fragment (ATF6f) (Hillary and FitzGerald, 2018). ATF6f is translocated to the nucleus and induces ERS factors (GRP78, XBP1), which are involved in cell death (Lakshmanan et al., 2013; Yang et al., 2015).

When the UPR occurs, the ER first tries to return to homeostasis by dampening the ERS, and if it is able to attenuate the UPR signal by reducing the number of misfolded proteins, the cell will survive; On the other hand, when homeostasis cannot be restored, the UPR signaling persists, activating apoptotic pathways and eventually leading to cell death (Oakes and Papa, 2015).

## 3 Endoplasmic reticulum stress and diabetic cardiomyopathy

**FIGURE 2:**
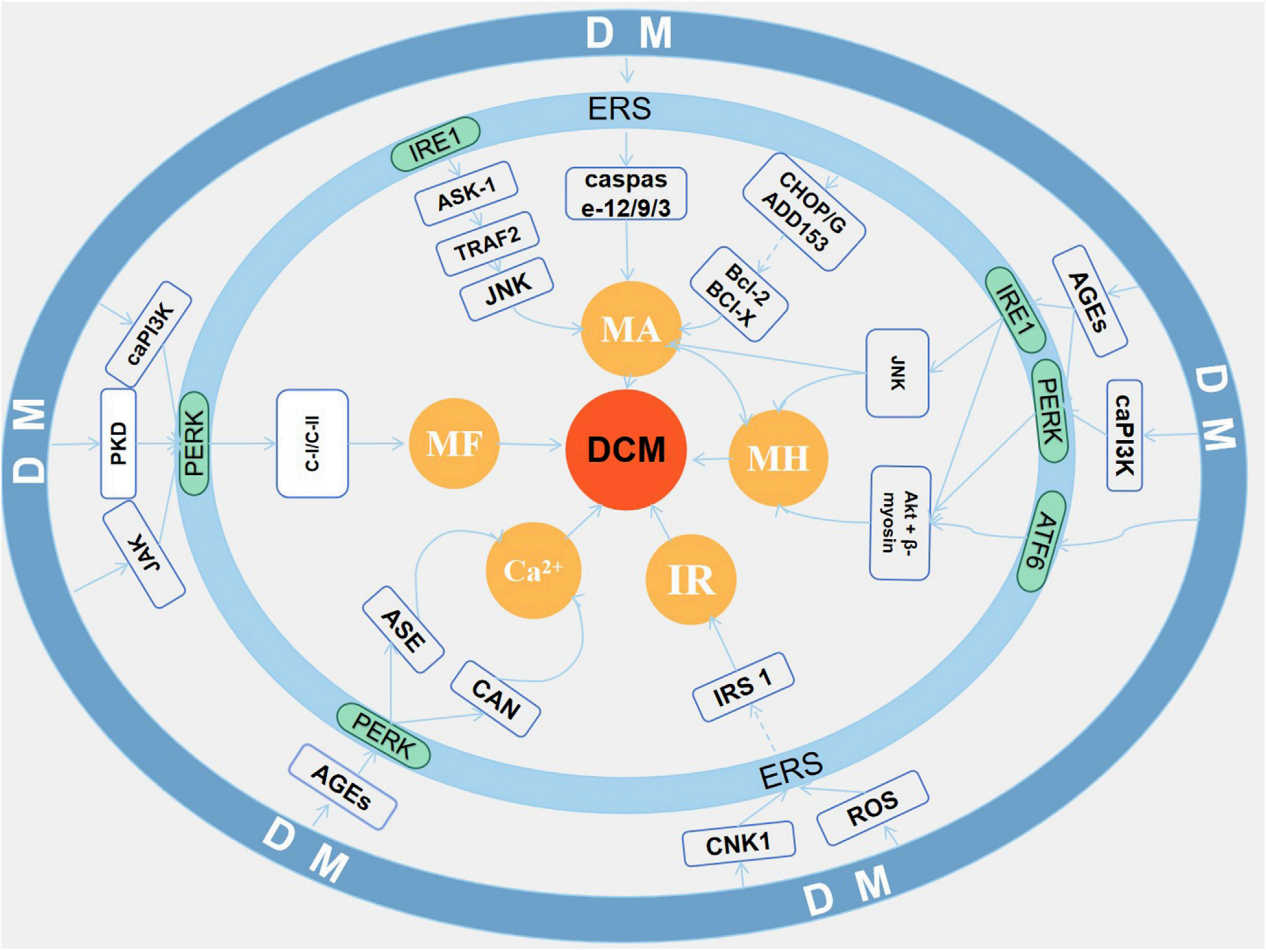
Endoplasmic reticulum stress and diabetic cardiomyopathy.

The pathogenesis of DCM is complex, and ERS is one of the important targets. In this study, databases (Pubmed, Web of Science and CNKI) were searched and a total of about 80 articles on research involving ERS mechanisms in DCM were found and finally summarized: ERS may be involved in the pathogenesis of DCM by affecting myocardial fibrosis, myocardial hypertrophy, cardiomyocyte apoptosis, myocardial insulin resistance, and disturbance of intracellular calcium regulation (as shown in Figure 2).

### 3.1 ERS and myocardial fibrosis

Myocardial fibrosis is one of the important pathological changes in DCM, including the accumulation of extracellular matrix proteins, particularly collagen, which associated with matrix metalloproteinase (MMPs) and MMP inhibitor function, involving sorbitol signaling pathway, diacylglycerol signaling pathway, etc (Asbun and Villarreal, 2006). In both diabetic patients and diabetic animal models, the manifestation of myocardial fibrosis has been observed in the diabetic heart.

It was shown that the T1DM rat model had reduced ejection fraction, elevated type III procollagen and laminin, increased expression of type I collagen, type III collagen and MMP-2/-9, wavy myocardial fibers and significantly increased collagen deposition, suggesting myocardial fibrosis and consequent cardiac dysfunction in this rat; further *in vitro* experiments showed that this phenomenon may be associated with activated ERS (increased expression of GRP78, GRP94, CHOP and ATF-4) (Hu et al., 2020). In addition, activation of ERS (increased expression of EIF2α, GRP94, and caspase-3) by Janus kinase and transcriptional activator signaling pathways resulted in disorganized cardiomyocyte arrangement and increased extracellular matrix collagen deposition in diabetic rats, which are involved in the process of myocardial fibrosis (Liu et al., 2018). Darnel Prakoso (Prakoso et al., 2020) et al. found that constitutively active phosphatidylinositol 3-kinase (caPI3K) is an important protein of ERS involved in myocardial fibrosis, and they found that decreased expression of caPI3K in DCM rats led to an increase in the expression of ERS-associated proteins GRP94 and CHOP, and an increase in the myocardial content of collagen types I and III and in the expression of connective tissue growth factor (CTGF), transforming growth factor-β (TGF-β), and tissue inhibitor metalloproteinase-2 (TIMP-2) expression, resulting in a significant increase in left ventricular interstitial and perivascular fibrosis. Gene therapy with a recombinant adeno-associated viral vector of the phosphatidylinositol 3 kinase construct (rAAV6-caPI3K) reduced ERS, decreased myocardial fibrosis, and improved myocardial remodeling. In addition, protein kinase D (PKD) is also an important target of ERS involved in cardiac remodeling, and PKD expression was elevated in the DCM rat model and the expression of the ERS chaperone molecule GRP78 was increased, and by correlation analysis, GRP78 was significantly correlated with PKD expression (r = 0.914) (Liu et al., 2015).

### 3.2 ERS and myocardial hypertrophy

Myocardial hypertrophy is another of the distinct pathological changes seen in DCM. Oxidative stress, insulin resistance and hyperglycemia are the reasons for the promotion of hypertrophic gene expression in cardiomyocytes (Grubic Rotkvic et al., 2021). In the case of cardiac hypertrophy, studies (Prakoso et al., 2020) have shown that caPI3K is also an important signaling pathway involved in ERS. After rAAV6-caPI3K treatment, cardiac natriuretic gene expression can be decreased, and cardiomyocyte width and cross-sectional area can be reduced, and the mechanism may be related to the reduction of ERS by rAAV6-caPI3K. The involvement of advanced glycosylation end products (AGEs) in the progression of ERS-induced DCM was demonstrated by Pei et al. (2018a). In STZ-induced DCM mice, cardiomyocyte cross-sectional area was increased and cardiac function was impaired; their increased phosphorylation of pro-hypertrophic signaling molecules Akt and mTOR correlated with increased phosphorylation of ERS signaling molecules GADD153, EIF2α, and IRE1α, which were ameliorated by aminoguanidine, an AGE inhibitor. It was shown that in HFD-induced DCM mice, the increase in heart weight to tibia length ratio indicates cardiac enlargement, and the increase in cardiomyocyte surface area and cross-sectional area indicates cardiac hypertrophy. The probable mechanism is that ERS initiation activates IRE1, and activation of IRE1 endonuclease activity shears XBP1 to s-XBP1 and activates the JNK pathway, inducing apoptosis, which in turn leads to pathological cardiac hypertrophy (Barr et al., 2015). Jia-hui Tian (Tian et al., 2021) et al. demonstrated that increased cardiomyocyte cross-sectional area, HG-induced increase in cardiomyocyte diameter, and markedly elevated levels of the hypertrophic markers cardiac natriuretic peptide and β-myosin heavy chain mRNA were associated with ERS-activated apoptotic pathways induced by ERS activation (GRP78, XBP-1S, ATF6, P-PERK, ATF4, CHOP) in DCM mice.

### 3.3 ERS and cardiomyocyte apoptosis

Apoptosis is a form of cardiomyocyte death, also known as programmed cell death, which is manifested by cytoplasmic shrinkage, nuclear enrichment, nuclear overflow, DNA fragmentation, plasma membrane blistering, and finally the formation of apoptotic bodies, and it is the fastest form of cell death; Among them, the protein cascade reaction induced by cysteine-induced aspartate protein hydrolase is a key feature, which amplifies the apoptotic pathway and leads to irreversible cell death (Chen et al., 2020). There are three pathways of ERS-induced apoptosis ERS (Xu et al., 2012). One of them is the c-Jun N-terminal kinase (JNK) signaling pathway, during ERS, activated IRE-1 recruits tumor necrosis factor-related factor 2 (TRAF2) and apoptosis signal-regulated kinase 1 (ASK-1) to activate JNK and downstream signals, in which c-Jun N-terminal inhibitory kinase (JIK) may be involved in regulating this pathway. The second pathway is the caspase-12 pathway, which is activated by multiple pathways during ERS, followed by cleavage of caspase-9 and activation of caspase-3, leading to apoptosis. The third pathway is mediated by transcriptional activation of CHOP/GADD153, which may be associated with enhanced apoptosis by inhibiting the expression of anti-apoptotic Bcl-2 and Bcl-X. In DCM rat hearts, TUNEL staining showed an increase in apoptotic cells in DCM hearts, decreased expression of anti-apoptotic protein Bcl-2, and significantly increased ERS-related proteins GRP78, caspase-9, and caspase-12; In addition, *in vitro* experiments significantly increased the expression of GRP78, IRE-1α, and CHOP in H9c2 cells cultured in HG; all suggesting that ERS may contribute to DCM development through the apoptotic pathway in DCM (Park et al., 2021). In addition, ERS is also involved in apoptosis in DCM through the CHOP and caspase-12 pathways, and this effect can be ameliorated by exercise (Chengji and Xianjin, 2019). Mitochondrial fusion protein-2 (MFN-2), a key protein linking mitochondria and endoplasmic reticulum, can induce cardiomyocyte apoptosis by mediating oxidative stress through ERS and mitochondrial apoptotic pathways (Yang et al., 2017).

### 3.4 ERS and myocardial insulin resistance

In addition to the common insulin receptor organs such as liver, fat, and muscle, the insulin signaling pathway is also present in the myocardium. Similar to the three tissues mentioned above, insulin signaling in the heart stimulates GLUT4 translocation to the cardiac cell membrane for glucose uptake via the insulin-insulin receptor substrate (IRS)-PI3K-protein kinase B (AKt) pathway (Jia et al., 2016). Myocardial insulin resistance impairs PI3K/Akt signaling, reduces glucose oxidation, increases intracellular Ca^2+^, and additionally activates endothelial NOS and reduces NO, leading to the development of DCM (Salvatore et al., 2021). Cannabinoid receptor gene (CNR1) is one of the important targets, Pei et al. (2018b) demonstrated that CNR1 expression was increased in HFD-induced mice with myocardial insulin resistance (decreased expression of IRS1/AMPKα/ACCA) and ERS, and further studies showed that knockdown of CNK1 ameliorated cardiac dysfunction and myocardial apoptosis in mice with DCM, and importantly, ameliorated insulin resistance and decreased ERS, and the *in vitro* studies further demonstrated that knockdown of CNK and activation of AMPKα ameliorated insulin resistance, suppressed ERS, and relieved DCM. Guanghong Jia and others concluded that in diabetic cardiomyopathy, in the presence of insulin resistance in the heart, nutrient overload leads to the production of ROS, and the excess of ROS disrupts protein folding in the rough endoplasmic reticulum, triggering ERS(68).

### 3.5 ERS and disturbance of intracellular calcium regulation

There is a special endoplasmic reticulum in heart muscle cells called the sarcoplasmic reticulum (SR) that is involved in the contractile function of the heart muscle. Impaired contractile function in DCM is associated with impaired SR function, which leads to a dysregulation of intracellular Ca2+ regulation, and the main mechanisms include the endoplasmic reticulum Ca2+-ATPase (SERCA2A) pathway (Trost et al., 2002). Furthermore, arrhythmic manifestations are common in DCM patients, and their mechanism is related to ERS. Zhongwei Liu (Liu et al., 2014) et al. showed that in DCM rats treated with ERS inhibitor 4-phenylacetic acid, the expression of ERS chaperone GRP78 was reduced, which inhibited ERS and decreased the number of premature beats, again proving that ERS was associated with arrhythmias in DCM, and further studies showed that PERK was a mediator; Using RNAi technology, the team confirmed that PERK in ERS could increase the activity of calmodulin neuralphosphatase, which induced increased Ca2+ in cardiomyocytes and led to arrhythmias. In addition, *in vivo* experiments in one study showed disturbed intracellular Ca2+ regulation in cardiomyocytes from DCM mice, while *in vitro* experiments showed that methylglyoxal-derived AGE (Mg-AGE) promoted oxidation of the calcium-regulated protein SERCA2a and disturbed intracellular Ca2+ homeostasis in isolated cardiomyocyte cultures, and that the above changes could be reversed by use of the ERS chaperone TUDCA(60).

## 4 TCM modulates ERS for DCM

### 4.1 Active ingredient in Chinese medicine

#### 4.1.1 Resveratrol (RES)

RES is a natural polyphenolic compound found in herbs such as thuja and cassia (Hou et al., 2019), with a variety of pharmacological effects including anti-inflammatory (Wang et al., 2018),anti-oxidative stress (Xu et al., 2016), nerve protection (Zhang et al., 2020), cardiovascular protection and more (Kaya et al., 2019; Zhang et al., 2022). Studies have shown that RES reduces the heart/body weight ratio, ameliorates myocardial hypertrophy, and improves cardiac function in DCM rats induced by a high-fat diet (HFD) combined with low-dose streptozotocin (STZ); in addition, RES, through PERK/eIF2α, ATF6/CHOP, and IRE1α/JNK-regulated ERS, reduced AGEs-induced myocardial apoptosis in H9C2 cells *in vitro* (Guo et al., 2015).

#### 4.1.2 Puerarin (PUE)

PUE, a member of the isoflavonoid group of compounds, is the main active ingredient extracted from Lobed Kudzuvine Root. Pharmacological studies have shown its pharmacological effects such as anti-oxidative stress (Zhou et al., 2022), anti-inflammation (Zhou et al., 2022), improvement of insulin resistance (Xu et al., 2021), cardioprotection (Xu et al., 2016), neuroprotection (Zhou et al., 2014). Ma Weibin et al. used high-fat and high-sugar feeding combined with intraperitoneal injection of STZ (35 mg/kg) to prepare diabetic rats with geranylgeranyl intervention, and the results showed that PUE can lower blood glucose, reduce insulin resistance index, inhibit cardiomyocyte apoptosis, ameliorate myocardial fibrosis, and have a protective effect on the myocardium of DCM rats, as shown by Ma Weibin et al. (Ma et al., 2012a; Ma et al., 2012b; Ma et al., 2013). The mechanism may be to attenuate the ERS by reducing the expression of GRP78, CHOP, caspase-3, and caspase−12.)

#### 4.1.3 Astragaloside IV(AS-IV)

AS-IV is one of the active constituents of the traditional Chinese medicine Membranous Milkvetch Root, which has a variety of pharmacological effects, including anti-oxidant stress (Xing et al., 2021),anti-inflammation (Feng et al., 2021),anti-ischemic brain damage (Xiao et al., 2021), and alleviation of fibrosis (Yu et al., 2022).Zhang Xiaofeng et al. (Zhan et al., 2022) used high glucose to induce a myocardial injury model of rat H9c2 cardiomyocytes and observed the effects of astragaloside in combination with geranylgeranyl on cardiomyocyte damage. Compared with the model group, the expression of GRP78, IRE1α, CHOP, XBP1u, XBP1s, and p53 upregulate modulator of apoptosis (PUMA) was decreased and the Bax/Bcl-2 ratio was decreased in the AS-IV combined with PUE group, which showed that the treatment of AS-IV combined with PUE could inhibit the high glucose-induced ERS and the related apoptotic effect on the ER in H9c2 cells, and thus alleviate the high glucose-induced damage to the cardiomyocytes.

#### 4.1.4 Mangiferin (MAN)

MAN (1,3,6,7-tetrahydroxyxanthone-C2-β-d glucoside) is a polyphenolic compound that is widely found in plants of the Anacardiaceae and Gentianaceae families. These plants include the herbs Mango, Anemarrhenae Asphodeloides, Chinese Gentian and so on (Gold-Smith et al., 2016). It has anti-oxidant, anti-virus, anti-tumor, anti-inflammatory, gene regulation, and hepatoprotective effects (Yang et al., 2020). Myocardial enzymes and inflammatory factors were reduced in DCM rats after MAN treatment (Hou et al., 2016), and cumulative reduction of myocardial collagen and improvement of myocardial fibrosis in DCM rats indicated that MAN had myocardial protective effect in DCM rats (Hou et al., 2013). A diabetic model was established by a single intraperitoneal injection of 45 mg/kg STZ in 230 g–280 g SSD rats, and different doses of mangiferin were administered by gavage for 12 weeks after successful modeling, which showed that MAN could ameliorate hyperglycemia, lower left ventricular systolic pressure, and reduce apoptosis rate in DM group rats; the mechanism may be related to MAN reducing cardiomyocyte IRE1, ASK1, and JNK protein expression and attenuating ERS(96).

#### 4.1.5 Ginseng fruit saponins (GFS)

GFS is a ginsenoside analog isolated from the ripe fruit of the traditional Chinese medicine ginseng, which improves insulin resistance (Luo et al., 2005) and protects the nervous and cardiovascular systems (Liu et al., 2016). GFS is also used in the treatment of stroke, Alzheimer’s disease, Parkinson’s disease, hypertension and myocardial ischemia due to its vasodilating, antioxidant and anti-inflammatory properties (Liu et al., 2019). Zhao Liang-chen et al. (Zhao et al., 2014) applied GFS to treat high-fat-high-glucose (HFHG) combined with STZ-induced DCM rats, and the results showed that GFS improved blood sugar, cardiac enzyme profiles, cardiac myocyte abnormalities, and cardiac myocyte apoptosis in DCM rats, which may be related to the fact that GFS decreased the expression of caspase-12 protein in cardiac myocytes. This experiment suggests that GFS can reduce ERS-induced apoptosis and protect cardiomyocytes.

#### 4.1.6 Ginsenoside Rg1(G-RG1)

Ginsenoside, a triterpenoid saponin compound, is the main active ingredient of traditional Chinese medicine ginseng, with anti-tumor, antioxidant and anti-inflammatory effects, widely used in cardiovascular, endocrine and immune system diseases (Yu et al., 2021). G-Rg1 is one of them. Yu Hai-tao et al. (Yu et al., 2016) used high-fat and high-sugar-fed rats for 1 month, induced T2DM by intraperitoneal injection of STZ, and treated with G-Rg1 at high, medium, and low doses for 3 months to observe its effect on the hearts of T2DM rats; G-Rg1 can reduce cardiac troponin-I., reduce the rate of apoptosis of cardiomyocytes, and reduce fibrosis; and it can reduce GRP78, CHOP, cleaved caspase-12, cleaved caspase-3, and elevated Bcl-cl. This suggests that G-Rg1 is indeed able to protect the heart of DCM rats, and its mechanism may be related to the attenuation of ERS in cardiomyocytes by ginsenoside Rg1.

#### 4.1.7 Naringin (Nar)

NAR is a flavanone glycoside, a natural flavonoid compound widely found in grapes and citrus fruits (Ravetti et al., 2023). NAR is the main active ingredient of the Chinese herbal medicines Citri Grandis Exocarpium, Aurantii Fructus Immaturus and Aurantii Fructus. Modern pharmacological studies have shown anti-inflammatory, antioxidant, anticancer, antibacterial and cholesterol-lowering effects (Wu et al., 2021). NAR improves basal glucose transport and lowers blood glucose, attenuates cardiac injury and increases cell viability in T2DM cardiomyocytes, while decreasing ROS production and reducing oxidative stress in the myocardium (Uryash et al., 2021). Further studies showed that Nar could reduce MAD levels and SOD activity in the myocardium of DM rats (modeling by single intraperitoneal injection of STZ at 60 mg/kg), while decreasing the expression of GRP78, CHOP, and caspase-12 proteins, inhibit ERS and oxidative stress, attenuate myocardial cell apoptosis, and reduce myocardial fibrosis in DCM rats (Zhang et al., 2018). However, the crosstalk relationship between oxidative stress and ERS needs further investigation.

#### 4.1.8 Curcumin (CUR)

CUR is a lipophilic polyphenolic compound extracted from turmeric, the main active ingredient of turmeric. It has pharmacological effects such as anti-inflammatory, antioxidant, improvement of glucose homeostasis, regulation of lipid metabolism, improvement of endothelial function and insulin signaling (Kim and Clifton, 2018). *In vitro*, CUR reduced the expression of ERS proteins (CHOP and GRP78) in palmitic acid (PA) or thapsigargin(THA) induced H9C2 cells, decreased caspase-3 activity, reduced BAX protein expression, and decreased the rate of PA or THA induced apoptosis, demonstrating that CUR protects H9C2 cells from lipotoxicity by ameliorating ERS(108). However, natural curcumin is poorly absorbed, so Yonggang (Wang et al., 2014) synthesized a turmeric analog, C66, from natural turmeric. They treated STZ-induced DCM mice with C66 and demonstrated that C66 could alleviate ERS and improve cardiac fibrosis and apoptosis in DCM mice (C57BL/6J mice, 8–10 weeks old, were selected and modeled by a single intraperitoneal injection of 150 mg/kg STZ. Blood glucose greater than or equal to 250 mg/dL 3 days after injection was used as the modeling criterion) through the IRE-1/ATF4/CHOP/csapase-12 pathway.

#### 4.1.9 Matrine (MAT)

MAT is a tetracyclic quinoline alkaloid, which is the main active ingredient of the traditional Chinese medicine Lightyellow Sophora Root, and also the main ingredient of composite Lightyellow Sophora Root injection; modern research has shown that MAT has the effects of anticancer, anti-fibrosis, anti-osteoporosis, anti-inflammation, anti-apoptosis, and so on (Wu et al., 2021) (Liu et al., 2017) used an STZ-induced DCM rat model (SD rats were injected intraperitoneally with 60 mg/kg STZ twice, and the criterion for modeling was non-fasting blood glucose >16.7 mmol/L for 2 consecutive postinjection sessions) with concomitant intervention with MAT, which was found to attenuate cardiac diastolic function, improve cardiac compliance, and inhibit cardiac fibrosis by attenuating the ATF6/calmodulin/NFAT signaling pathway in the heart in DCM rats; the results suggest that MAT inhibits ATF6 signaling in the ERS as well as its downstream calponin and NFAT pathways, thereby treating DCM. Huijuan and others concluded used MAT intervention in STZ-induced DCM and cardiomyocytes isolated from DCM, and found that MAT could suppress PERK expression and reduce cardiomyocyte apoptosis in DCM rats, indicating that MAT could suppress ERS to achieve therapeutic effects in DCM (Hou et al., 2019).

#### 4.1.10 Astragalus polysaccharide (APS)

APS is one of the main active ingredients extracted from the traditional Chinese medicine Astragalus, and its main pharmacological effects include immune regulation, anti-aging, lowering blood glucose, lowering blood lipids, anti-fibrosis, anti-bacterial, anti-radiation, anti-viral and so on (Zheng et al., 2020). Sun et al. demonstrated that APS improved LVEDD, LVESD, left ventricular end-diastolic volume (LVEDV), left ventricular end-systolic volume (LVESV), fractional shortening (FS), and LVEF indices in DCM rats (after 1 week of acclimation feeding, 200–250 g SD male rats were given a single intraperitoneal injection of 60 mg/kg STZ and those with blood glucose levels >16.7 mmol/L 1 week after injection were included in the study.); and reduced apoptosis in high glucose-cultured H9C2 cells due to inhibition of PERK and ATF6 pathways in ERS by APS (CHOP, p-PERK, p-JNK, p-IRE1α, and caspase-12) (Sun et al., 2019).

#### 4.1.11 Tanshinone IIA (TS-IIA)

TS-IIA (Molecular Formula: C19H18O3, MW: 294.3), is a diterpenoid quinone from S. miltiorrhiza with red needle crystal (Zhong et al., 2021). TS-IIA has pharmacological effects such as anti-tumor, anti-oxidative stress, anti-inflammation, anti-fibrosis, inhibiting apoptosis, inducing autophagy and improving atherosclerosis (Zhang et al., 2019; Wang et al., 2020; Ding et al., 2020; Fang et al., 2020; Bi et al., 2021; Lu et al., 2022). TS-IIA upregulates miroRNA-133 to ameliorate ERS-mediated apoptosis to protect cardiomyocytes (Feng et al., 2016). Further studies showed that TS-IIA alleviated pathological changes in the hearts of diabetic mice (6-week-old SD rats, single intraperitoneal injection of 60 mg/kg STZ, 2 days post-injection, with blood glucose greater than 16 mmol/L as the criterion for modeling) and inhibited the expression of ERS-related proteins and mRNAs (GRP78, CHOP), suggesting that Tan IIA ameliorates DCM by inhibiting ERS (Tao et al., 2019; Wu et al., 2023).

### 4.2 Chinese herb

#### 4.2.1 White mulberry root-bark (WMR)

WMR has the function of removing heat from lung and relieving asthma, inducing diuresis to alleviate edema. WMR, containing flavonoids, alkaloids and stilbenoids, has antimicrobial, skin-whitening, cytotoxic, anti-inflammatory, and anti-hyperlipidemic properties (Chan et al., 2016). Jiangfang et al. (Lian et al., 2017) used the lyophilized powder of WMR to study its protective effect and mechanism on STZ-induced DCM myocardium(250–300 g SD male rats fed high-fat chow for 8 weeks were added to 25 mg/kg STZ injection, and those with blood glucose >16.7 mmol/L at 1 week post-injection were included in the study), and the results showed that WMR could inhibit the expression of PERK and its phosphorylation, and reduce the expression of eIF2α, ATF4, and CHOP; it was proved that WMR could inhibit ERS and protect the cardiac function of DCM by inhibiting the PERK/eIF2α/ATF4/CHOP pathway.

#### 4.2.2 Ginkgo biloba leaves (GBL)

GBL is widely used to treat diabetic complications, cardiovascular disease, Alzheimer’s, vascular dementia and so on. The components of Ginkgo biloba leaf extract (GBE) contain many constituents, such as terpene trilactones, flavonoids, phenolic acids, amino acids, saccharides, and various organic acids (Horbowicz et al., 2022). GBE has anti-inflammatory, improved innate immunity, anti-apoptotic, antioxidant, anti-fibrotic, and gut microbiota-regulating activities (Kim et al., 2021; Mousavi et al., 2022; Sochocka et al., 2022; Wei et al., 2022; Akanchise and Angelova, 2023). In treating DCM, GBE significantly attenuated cardiomyocyte apoptosis, collagen deposition, and inflammatory responses in HFD(basic diet, 78.85%; fat, 21%; and cholesterol,0.15%) combined with STZ(50 mg/kg/day, 5 days)-induced ApoE^−/−^ mice by inhibiting p-JNK, CHOP, and caspase-12 pathways (Tian et al., 2018). In the treatment of diabetic myocardial injury, GBE may be beneficial.

## 5 Discussion and conclusion

DCM has a high morbidity and mortality, which seriously affects the quality of life of patients and increases the burden on society. A prospective study showed that after excluding a history of structural heart disease, 100 diabetic patients were included, 48% of whom had DCM, and after a mean follow-up of 48.5 ± 9.0 months, patients with DCM experienced more deaths or cardiovascular events than DM patients without DCM (12.5% vs. 3.9%) (Kiencke et al., 2010). Therefore, it is critical to explore the pathogenesis of DCM. However, the pathogenesis of DCM is complex. Recent studies have identified aldose reductase as a novel target for the development of DCM (Gopal et al., 2023). Phase 3 randomized, placebo-controlled, double-blind, global clinical trial of aldose reductase inhibitors in heart failure due to DCM is now underway, with promising clinical applications (Januzzi et al., 2023). In addition, a potential target for the diagnosis, prognosis and treatment of DCM is expected to be the unique expression profile of microRNA (miR) in the development of DCM (Rhman and Owira, 2022). For example, miR-29b-3 regulates the expression of Sarcolemmal membrane-associated protein (Fang et al., 2023), miR-18a-3p regulates the expression of gasdermin D (Yang et al., 2023), miR-340-5p regulates the expression of myeloid cell leukemia 1 (Zhu et al., 2022), and miR-126 regulates the expression of nuclear Erythroid2-related factor-2 (Asgari et al., 2022), all of which modulate DCM.

ERS is another important mechanism in the development of DCM and affects a wide range of DCM pathologic processes. A large number of studies have shown that ERS is closely related to DCM. By searching the database and summarizing, we concluded: in DCM, hyperglycemia, oxidative stress, and AGEs induce the development of ERS, which leads to myocardial fibrosis, myocardial hypertrophy, myocardial insulin resistance, myocardial cell apoptosis, and disturbances in the intracellular regulation of calcium ions. Ultimately, this leads to myocardial cell injury, which promotes the development of DCM. Current studies have focused on exploring specific targets of ERS-regulated DCM and preliminary exploration of drug therapy, and found that irisin (Li et al., 2022), melatonin (Huang et al., 2022), LCZ696 (Belali et al., 2022), etc. may treat DCM through anti-ERS, but lack of evidence for further clinical studies. Inhibitors of ERS are currently showing promising results in diabetic complications and cardiovascular disease. GSK, a PERK inhibitor, reduces mortality and arrhythmia risk in mice after myocardial infarction (Liu et al., 2021); Proteotoxicity inhibitor 4-PBA attenuates high glucose-induced DCM (Deng et al., 2022). IRE1 inhibitor STF-083010 regulates retinal endothelial inflammatory process under high glucose conditions (Papandreou et al., 2011). IRE1 inhibitor MKC-3946 reduces apoptosis of vascular smooth muscle cells to reduce aortic dissection (Zhang et al., 2022). As can be seen, there are many small molecules that can inhibit endoplasmic reticulum stress, but there is a lack of clinical trial evidence for their safety and efficacy.

In the present study, the beneficial effects of herbal medicines against ERS for the prevention of DCM were also found. Targeting ERS is a potential direction for combating DCM. It was found that the active ingredients of Chinese herbs (RES, PUE, AS-IV, MAN, GFS, G-Rg1, NAR, CUR, MAT, APS, TS-IIA) and Chinese medicines (WMR, GBL) could improve cardiac dysfunction caused by DCM and alleviate the disease progression by modulating endoplasmic reticulum stress (as shown in Table 1). However, there are still some problems in studying the mechanism of DCM modulation by targeted ERS TCM (Jia et al., 2018): Nowadays, the studies on ERS are mostly on a few representative proteins of ERS, involving more myocardial apoptotic pathways and fewer other pathways, which lacks the depth of research, and can be further investigated in depth in the direction of ERS to improve insulin resistance and intracellular calcium homeostasis imbalance (Rub et al., 1972); In the preclinical model of DCM, HFD combined with STZ injection is mostly used to induce (as shown in Table 1), and the drug intervention is mostly to prove the T2DM model before the drug intervention, and there are few descriptions about the DCM model, all of them prove that the DCM model is established after the end of treatment, which cannot make clear whether the role of drug intervention is a therapeutic role or preventive role; In the future, to determine whether it is a model or not, we can randomly sample the indices such as LVSP, LVEDP, and +dp/dtmax before the drug intervention (Macvanin et al., 2023); TCM therapy has multi-pathway and multi-target characteristics, the current study mostly indicates that the therapeutic effect is related to ERS, but cannot fully prove that it is through ERS to play a role in the lack of control group settings, so future studies can add ERS activators or inhibitors as a control group to further validate (Seferovic et al., 2018); The purpose of basic research is to translate it into clinical practice. Currently, most of these studies focus on animal or cellular models, and there is a lack of evidence from clinical trials; follow-up studies require high-quality, randomized, controlled, double-blind, evidence-based research.

**TABLE 1:** The effect of traditional Chinese medicine targeting ERS in the treatment of DCM.

Category	Compounds	Intervention objects	Experiment model	Result	Molecular mechanism	Reference
Active ingredient in Chinese medicine	RES	SD rats	HFD + STZ	Weight↓, LVPW↑, IVS↑, EF↑, myocardial apoptosis↓	SERCA2α↑,PERK/EIF2α↓, ATF6/CHOP↓, IRE1α/JNK↓, Caspase-12↓, Caspase-3↓, and Caspase-9↓	Guo et al. (2015)
		H9C2 cells	AGES 400 ug/ml			
	PUE	Wistar rats	HFHD4w + STZ 35 mg/kg	FBS↓, CK↓, LDH↓, myocardial morphology↑	GRP78↓, CHOP↓, Caspase-12/Caspase-3↓	Ma et al. (2012a), Ma et al. (2012b), Ma et al. (2013)
	AS-IV	H9C2 cells	35 mmol/L D- glucose	cell viability↑, apoptosis↓	GRP78, IRE1α, CHOP, XBP1u, XBP1s, PUMA, Bax/Bcl-2	Zhan et al. (2022)
	MAN	SD rats	STZ 45 mg/kg	FBG↓, LVSP↑, LVEDP↓, + dp/dtmax↑, -dp/dtmax↑, myocardial apoptosis↓	IRE1↓, ASK1↓, JNK↓	Chen et al. (2015)
	GFS	Wistar rats	HFHG4w + STZ 40 mg/kg	FGB↓, TC↓, myocardial enzymes↓, myocardial apoptosis↓, myocardial morphology↑	Caspase-12↓	Zhao et al. (2014)
	G-Rg1	Wistar rats	HFHG4w + STZ 40 mg/kg	Weight↓, CTNI↓, LVEDD↓, E/A↓, LVPWD↓, myocardial apoptosis↓, myocardial fibrosis↓	GRP78↓, CHOP↓, Caspase-12↓,	Yu et al. (2016)
	NAR	SD rats	STZ 60 mg/kg	FBG↓, cardiac index↓, myocardial fibrosis↓	SOD↑, MDA↓, GRP78↓, CHOP↓, caspase-12↓	Zhang et al. (2018), Uryash et al. (2021)
	CUR	H9C2 cells	PA 400 mmol/L	cell viability↑, apoptosis↓	GRP78↓, CHOP↓, caspase-3↓, Bax↓	Guan et al. (2019)
		C57BL/6J mice	STZ 150 mg/kg	EF%↑, FS↑, LVPW↑, IVS↑, myocardial fibrosis↓	GRP78↓, CHOP↓, IRE-1↓, ATF4↓, caspase-3↓, caspase12↓, Bax↓, Bcl-2↓	Wang et al. (2014)
	MAT	SD rats	STZ 60 mg/kg (2 times in a row)	LVEDP↑, LVSP↓, myocardial fibrosis↓	ATF6/calmodulin/NFAT↓	Liu et al. (2017)
		SD rats	STZ	LVdP/dt max↑, LVdP/dt min↑, LVSP↑, LVEDP↓, myocardial apoptosis↓, inflammatory↓, Microvascular necrosis↓	PERK↓, TGF-β, CHOP↓, ATF-4↓, caspase-3↓, caspase-9↓, Bcl-2↓	Hou et al. (2019b)
		DCM rat cardiomyocytes	—			
	APS	SD rats	STZ 60 mg/kg	Glu↓,Weight↑, LVEDD↓, LVESD↓, LVEDV↓, LVESV↓, EF↑, FS↑, myocardial apoptosis↓, myocardial fibrosis↓	CHOP↓, p-PERK↓, p-IRE1α(−), p-JNK(−), Caspase-12(−)	Sun et al. (2019)
		H9C2 cells	HG 33 mM			
	TS-IIA	SD rats	STZ 60 mg/kg	Glu↓, mitochondrial density↑, mitochondria and myocardial morphology↑	GRP78↓, CHOP↓,SOD↑	Tao et al. (2019)
Chinese herb	WMR	SD rats	HFD8w + STZ 25 mg/kg	Glu↓, INS↓, TG↓, myocardial fibrosis↓, myocardial morphology↑	PERK/eIF2α/ATF4/CHOP↓	Lian et al. (2017)
	GBL	ApoE^−/−^ mice	HFD4w + STZ 50 mg/kg/d qd 5d	LDL-C↓, TC↓, TG↓, GLU↓, IL-6↓, myocardial fibrosis↓, myocardial morphology↑	Caspase-3↓, CHOP↓, p-JNK↓, Caspase-12↓	Tian et al. (2018)

Although there are some problems in studying ERS modulation by TCM for treating DCM, with the further development of science, ERS modulation by TCM may provide a new direction for treating DCM. The treatment of diabetes and cardiovascular diseases with Chinese medicines has a history of thousands of years and has achieved good efficacy in Chinese patients, but it is difficult to promote due to the cultural differences between the East and the West, the low quality of medicinal materials, the backwardness of the extraction process, the lack of science in the quality standards and the quality control system, the difficulty in pharmacokinetic studies, the difficulty in evaluating the efficacy of the treatment, the low level of systematization, and the insufficient protection of intellectual property rights (Zhang and Jiang, 2022). It is hoped that more researchers will join in to conduct further in-depth studies on the above issues to accelerate the modernization of Chinese medicine.Under the guidance of Chinese medicine theory, it is hoped that a more precise and personalized treatment plan for patients will be developed through the use of evidence-based Chinese medicine treatment in combination with basic Western medicine treatment. Chinese medicine has unique advantages in regulating ERS to improve DCM, improve the in-depth study of the mechanism of ERS regulation by Chinese medicine compounding, and excavate natural ERS inhibitors from Chinese medicine with complex composition, so as to provide theoretical basis for the research and development of new drugs, and new strategies for preventing and treating DCM by Chinese medicine.

## Abbreviations

ASE, ASERCA2A; CAN, calmodulin neuralphosphatase; C-I/C-II, type I/II collagen; Ca2+, disturbance of intracellular calcium regulation; IR, insulin resistance; MA, myocardial apoptosis; MH, myocardial hypertrophy; MF, myocardial fibrosis. Straight lines indicate facilitated activation, dashed lines indicate diminished inhibition.
